# Altered physiology of drug-resistant *Mycobacterium tuberculosis* restricts adaptation to host-relevant conditions and produces condition-dependent collateral vulnerabilities

**DOI:** 10.1128/mbio.00244-26

**Published:** 2026-09-24

**Authors:** William J. Jowsey, Kate R. Harding, Caitlin E. Cunniffe, Chen-Yi Cheung, XinYue Wang, Caitlan J. Smart, Hannah R. Klaus, Amanda L. Peterson, Brunda Nijagal, Mark B. Hampton, Gregory M. Cook, Matthew B. McNeil

**Affiliations:** 1 Department of Microbiology and Immunology, University of Otago 2495, Dunedin, New Zealand; 2 Metabolomics Australia, Bio21 Institute, The University of Melbourne, Melbourne, Australia; 3 Mātai Hāora–Centre for Redox Biology and Medicine, Department of Pathology and Molecular Medicine, University of Otago Christchurch 2494, Christchurch, New Zealand; 4 School of Biomedical Sciences, Queensland University of Technology, Translational Research Institute 373031, Woolloongabba, Queensland, Australia; 5 Department of Biochemistry, University of Otago 2495, Dunedin, New Zealand; University of Oxford, Oxford, United Kingdom

**Keywords:** *Mycobacterium tuberculosis*, drug resistance mechanisms, physiology, fitness

## Abstract

Antibiotic resistance mutations often disrupt essential cellular processes; however, the physiological consequences of these mutations under host-relevant conditions remain poorly understood in many bacterial pathogens. Here, we have used a combination of phenotypic growth assays and metabolomics to investigate how drug resistance affects the metabolism of *Mycobacterium tuberculosis* and its ability to adapt to different host-relevant conditions. This work shows that (i) the fitness costs of antibiotic resistance in *M. tuberculosis* are highly variable and can be exacerbated or alleviated depending on the media carbon source, (ii) genetically unrelated resistance mutations have overlapping phenotypes and metabolic dysregulation, (iii) the growth of bedaquiline- and rifampicin-resistant mutants was impaired on host-relevant carbon sources, and (iv) resistance mutants have condition-specific collateral antibiotic susceptibilities, with rifampicin-resistant mutants being hypersusceptible to ATP synthase inhibitors when grown under conditions that require propionate metabolism. Collectively, this study highlights how drug resistance impacts bacterial physiology and places metabolic constraints on the ability of *M. tuberculosis* to adapt to changes in host-relevant conditions. This work has major implications for our understanding of how resistance evolves within the host context and reinforces the importance of how physiological variation in *M. tuberculosis* might affect the outcomes of new treatment regimens.

**IMPORTANCE:**

Mutations that provide antibiotic resistance in bacterial pathogens often disrupt key cellular processes. The effects of these disruptions on cellular physiology are poorly understood, especially within the context of a host environment. In this article, we investigated how antibiotic resistance mutations affect the ability of *Mycobacterium tuberculosis*, the causative agent of tuberculosis, to grow under host-relevant conditions. Using techniques that characterize growth and metabolism, we demonstrate that antibiotic-resistant *M. tuberculosis* can have both increased and decreased growth depending on the media carbon source, highlighting how interactions between drug resistance traits and the environment affect the ability of *M. tuberculosis* to adapt to changing conditions. We also show that unrelated antibiotic-resistant strains share similar metabolic defects, which can be exploited to improve antimicrobial susceptibility. Overall, this work demonstrates that drug resistance reshapes the physiology of *M. tuberculosis* in growth-dependent ways, having major implications for our understanding of how drug resistance evolves within the host context and for the design of new treatment strategies against antibiotic-resistant *M. tuberculosis*.

## INTRODUCTION

*Mycobacterium tuberculosis* is an obligate human pathogen and a causative agent of tuberculosis. Inherent antibiotic resistance, immune system evasion, and the ability to evolve resistance to all clinical antibiotics have resulted in tuberculosis becoming the leading cause of death worldwide due to an infectious disease (1). Within a host, *M. tuberculosis* survives and proliferates under hostile conditions that are low in O_2_, rich in reactive oxygen and nitrogen intermediates, and nutrient limited, where host-derived lipids, pyruvate, and lactate are primarily used for energy generation (2–7). Despite this, research in mycobacterial physiology is often conducted in conditions optimized for growth, potentially masking condition-dependent phenotypes.

Antibiotic resistance is acquired in *M. tuberculosis* by mutations that alter the structure or expression of the primary antibiotic target, prevent activation of a drug, or result in overexpression of efflux pumps. Such drug resistance-conferring mutations commonly impact essential cellular processes and impose fitness costs that are commonly observed as growth defects, remodeling of the cell wall, altered protein abundances, decreased levels of phosphoenolpyruvate, and altered antibiotic sensitivities (8–14). However, prior reliance on optimized growth media leaves us with a poor understanding of how mycobacterial drug resistance affects physiology in a host-relevant context, and importantly, how host-relevant metabolic defects impact the evolution of resistance in a clinical setting.

Here, we have used a combination of phenotypic growth assays and mass spectrometry (MS) to investigate how the growth and metabolic flexibility of *M. tuberculosis* are dysregulated by mutations associated with the acquisition of drug resistance. We performed experiments under nutrient-limited conditions with host-relevant primary carbon sources, as we speculated that standardized growth media, which are rich in multiple carbon sources and redox-buffering components, would mask condition-dependent fitness defects. Our results show that the fitness costs of drug resistance on *M. tuberculosis* are highly variable and can be alleviated or exacerbated by the growth environment. This included impaired growth for multiple drug-resistant (DR) strains when grown with propionate or lactate and alterations in the levels of metabolites of central carbon metabolism. Finally, antibiotic efficacy is greatly impacted by growth conditions, with DR strains exhibiting condition-dependent collateral drug sensitivity phenotypes. Overall, this study illustrates that the impact of drug resistance is pleiotropic in nature, with resistance mutations altering mycobacterial physiology through interactions between the genome, metabolome, and environment.

## RESULTS

### Isogenic DR *M. tuberculosis* strains exhibit constrained metabolic flexibility

**Fig 1:**
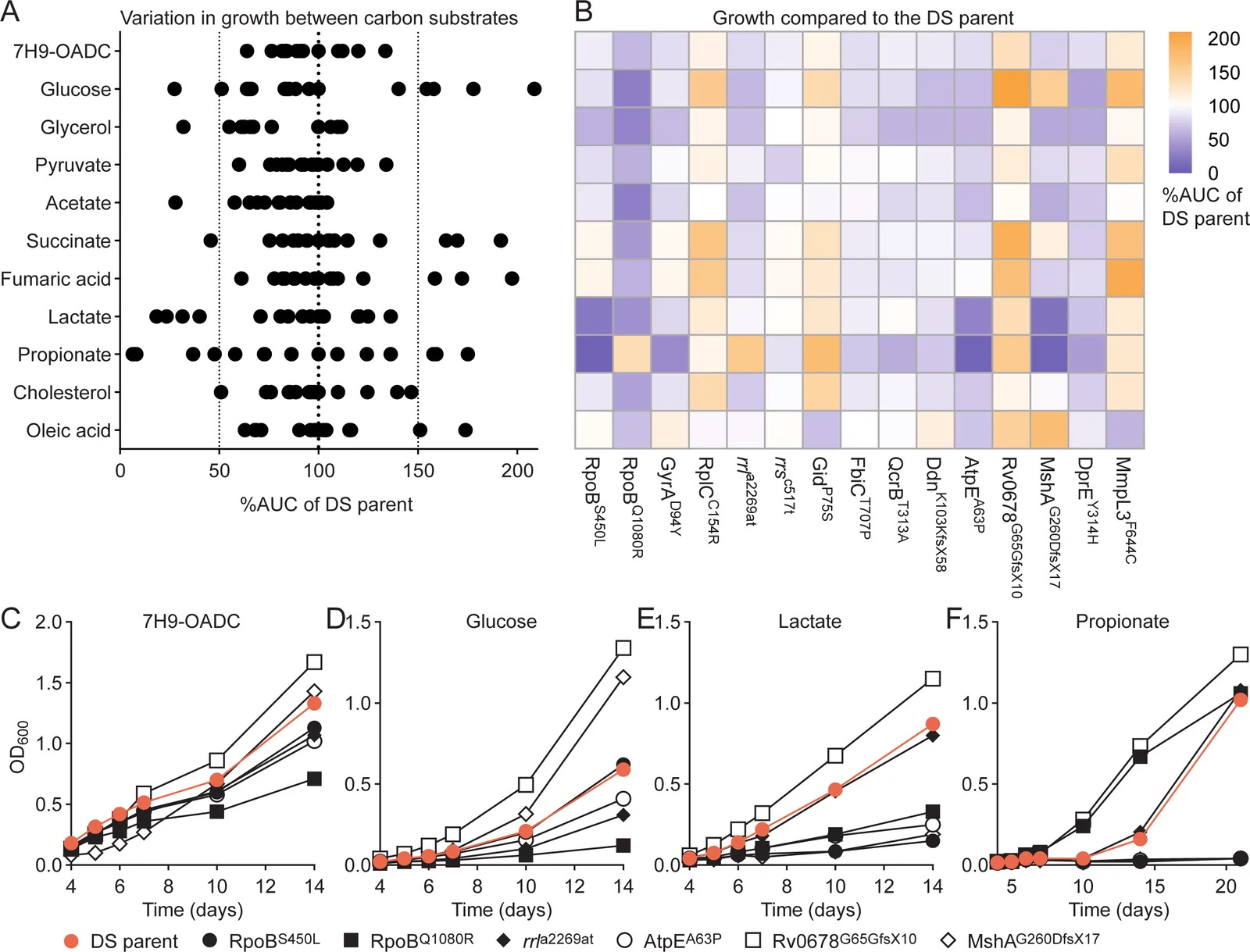
DR *M*. *tuberculosis* strains show diverse growth phenotypes in different carbon substrates. (**A**) The percentage of the mean AUC (overall growth across a period of time) for DR strains (closed black circles) in comparison to the DS parent strain (100%). Circles represent average values from at least two experiments, each comprised of at least two technical replicates. (**B**) Heatmap representation of panel **A**, with orange depicting higher growth (up to 210%) and blue lower (up to 0%). (**C–F**) Growth curves showing the growth of select DR strains on (**C**) 7H9-OADC and 7H12 with glucose (**D**), lactate (**E**), and propionate (**F**). Symbols show the average OD_600_ values from representative experiments. Strain symbols are depicted below panels **C–F**. Raw growth data, AUC, and µMAX values with associated statistical analyses for all conditions are presented in Table S1.

**Fig 2:**
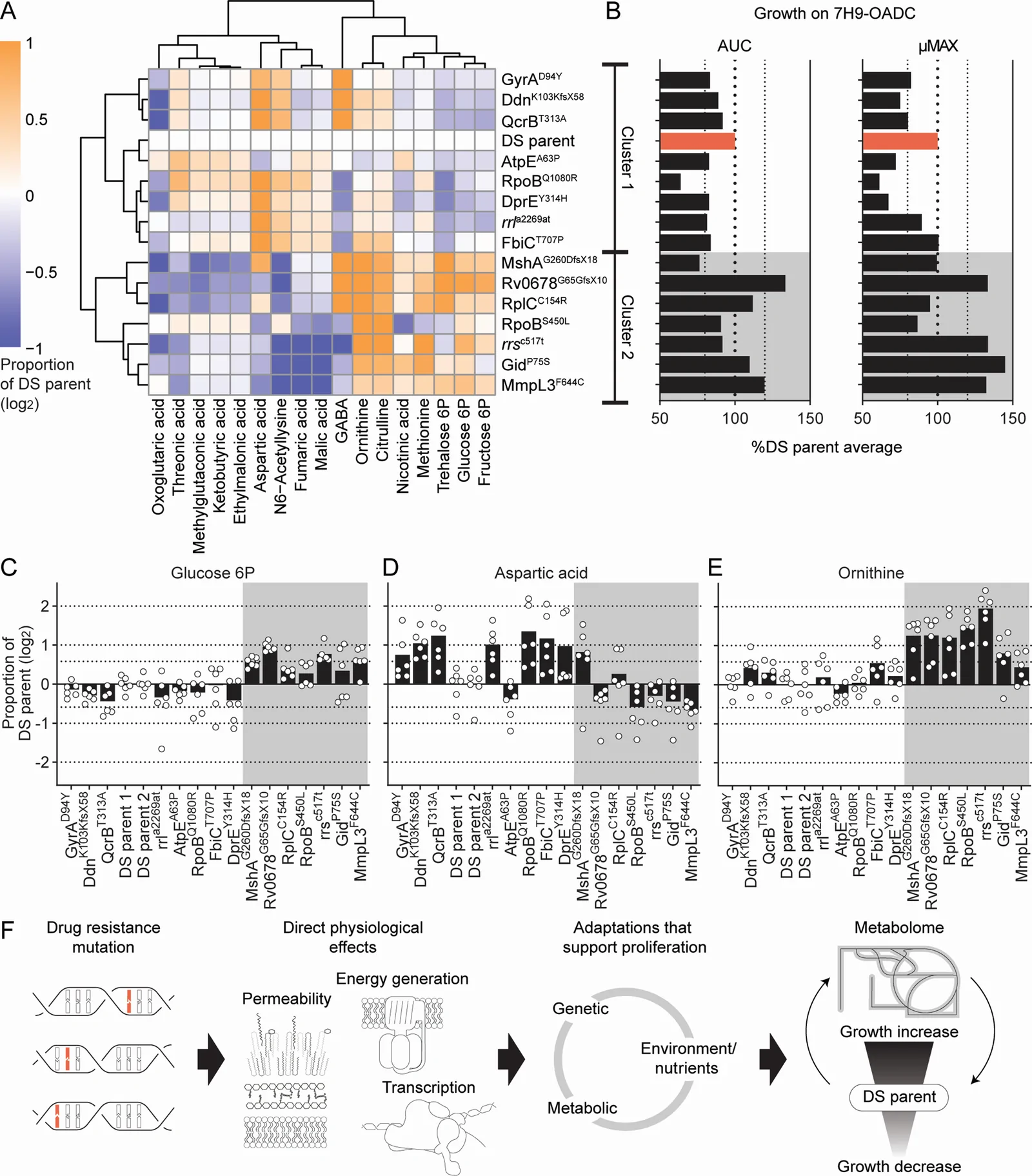
Metabolite levels of DR *M*. *tuberculosis* parallel growth differences. (**A**) Heatmap and hierarchical clustering of metabolites exhibiting significant differences between samples (*P* < 1 × 10^−5^ in the Kruskal-Wallis test with no multiple comparison testing correction; excluding sorbitol and gluconic acid, which featured strong outliers). Fold change (log_2_) is represented with orange depicting higher metabolite levels than the DS parent average and with blue for lower levels. Hierarchical clustering was performed using Euclidean distances and the Ward.D2 method. (**B**) Mean AUC and µMAX of DR strains compared to the DS parent average, with strains arranged to align with the clustering of panel **A**. Clusters one and two were distinguished based on the two major hierarchical clustering branches. (**C–E**) Average (black bars) and individual (open circles) metabolite levels of glucose 6-phosphate (**C**), aspartic acid (**D**), and ornithine (**E**) of the DR strains compared to the respective DS parent average. DS parent values are shown from both data sets. Dotted lines indicate the fold change (log_2_) from low to high: −2, −1, −0.5, 0.5, 1, and 2. Gray backgrounds demarcate cluster two strains. Raw and processed metabolite levels are presented in Table S2. Culture OD_600_ values at the time of extraction are presented in Table S3. (**F**) Summary model of how the metabolomes of DR strains compared to the DS parent are more a feature of growth than direct resistance mutations.

We hypothesized that resistance mutations in *M. tuberculosis* would impact growth under host-relevant conditions. To investigate this, we used a panel of 15 DR strains previously isolated in *M. tuberculosis* mc^2^6206 (13). We performed growth assays with the output area under the curve (AUC) to qualitatively measure overall growth and maximum growth rate (µMAX) to reveal the fastest growth rates. Consistent with previous reports that observed minimal fitness costs of drug resistance (13, 15), the DR *M. tuberculosis* strains showed comparable growth to the drug-susceptible (DS) parent (within 20% of the DS parent AUC) when grown in conventional 7H9-OADC liquid medium. The fidaxomicin-resistant mutant RpoB^Q1080R^ showed the least growth, while the bedaquiline-resistant Rv0678^G65GfsX10^ mutant showed the greatest (Fig. 1A through C). Analyses of growth rates revealed similar trends, but with more marked differences, including lower µMAX values (<80% of the DS parent) in strains carrying mutations in oxidative phosphorylation genes (e.g., AtpE^A63P^, Ddn^K103KfsX58^, and QcrB^T313A^) and in the strains RpoB^Q1080R^ and DprE^Y314H^ (Fig. 2B).

While OADC supplementation is common in *M. tuberculosis* growth media, it contains various carbon sources (glucose, oleic acid, glutamate, albumin, and glycerol in some cases) and components that can buffer oxidative stress (e.g., albumin [16] and catalase). We hypothesized that these components combined with the ability of *M. tuberculosis* to co-catabolize carbon substrates would mask the metabolic consequences of drug resistance. To investigate this, we performed growth assays in 7H12 media (17) that contained a single primary carbon source and lacked buffering components (i.e., 7H9 salts with just casamino acids and a carbon source). Overall, these media produced much greater variations in DR strain growth levels than did 7H9-OADC, with the largest variations in growth observed in 7H12 with glucose or propionate as the primary carbon source (Fig. 1A and B; Fig. S1). While growth differences between the DS parent and DR strains on glucose correlated with those observed for 7H9-OADC, various DR strains showed distinct growth impairment on propionate, including MshA^G260DfsX18^, RpoB^S450L^, AtpE^A63P^, and GyrA^D94Y^ (Fig. 1B, D, and F). These same strains also showed low growth on L-lactate (Fig. 1E). In contrast, RpoB^Q1080R^ showed increased growth on propionate despite showing consistently low growth on all other carbon substrates (Fig. 1B). Growth on gluconeogenic substrates (e.g., succinate) was favored by strains that had high growth on 7H9-OADC and glucose (e.g., RplC^C154R^ and MmpL3^F644C^), which contrasted with these same strains showing extended lag phases on fatty acids (e.g., oleic acid) (Fig. 1B; Fig. S2). Strains including MshA^G260DfsX18^, *rrs*^c517t^, and *rrl*^a2269at^ also showed extended lag phases on various carbon substrates (e.g., glycerol, pyruvate, and acetate) (Fig. S2), reflecting difficulties in adapting to these substrates compared to the DS parent. In conclusion, the fitness cost of drug resistance in *M. tuberculosis* is highly variable and condition-dependent, limiting the ability of DR strains to adapt and grow on different host-relevant carbon sources.

### Altered metabolism of DR strains parallels growth differences

We hypothesized that the constrained metabolic flexibility of DR strains was the result of resistance mutations having unintended effects on key metabolic pathways. To investigate this, intracellular polar metabolites were extracted from cultures grown to mid-late exponential phase in 7H9-OADC and compared to a set of metabolite standards. In total, 108 metabolites were identified across all samples (Table S2). Metabolites that showed the most significant variation among the strains included TCA cycle metabolites (e.g., oxoglutaric acid), amino acids (e.g., aspartic acid), and glycolytic intermediates (e.g., glucose 6-phosphate) (Fig. 2A), similar to the metabolic rewiring *M. tuberculosis* exhibits in response to different stressors (18). However, the extent of the differences in metabolite levels was typically <2-fold (Fig. 2A). Hierarchical clustering organized strains into two distinct clusters, which paralleled growth kinetics in 7H9-OADC (Fig. 2A and B). DR strains with lower AUC and/or µMAX values compared to the DS parent were placed in cluster one (e.g., AtpE^A63P^ and RpoB^Q1080R^, including the DS parent), while higher AUC/µMAX strains were in cluster two (e.g., Rv0678^G65GfsX10^ and MmpL3^F644C^) (Fig. 2B). RpoB^S450L^, despite being placed in cluster two, shared various metabolic differences with the slower growing cluster one strains (Fig. 2A). Additional metabolic differences identified in the untargeted MS data analyses supported how the strains were clustered (Fig. S3).

We hypothesized that metabolites with reciprocal levels of abundance between the two clusters represented metabolites that were drivers of different levels of growth. Cluster two strains showed higher abundances of phosphorylated mono- and di-saccharides (Fig. 2A and C), consistent with elevated growth on 7H9-OADC and 7H12-glucose media (Fig. 1B) and either increased uptake (19, 20) or greater carbon flux through glycolysis (21, 22). Aspartate, which links various metabolic pathways (e.g., TCA cycle, amino acid, and nucleoside biosynthesis), was mostly increased in slower-growing cluster one strains (Fig. 2A and D), indicating that these strains were more reliant on anaplerosis to sustain TCA cycle turnover (23, 24). Altered levels of N6-acetyllysine suggested that lysine acetylation was dissimilar between the clusters, which supported differences in central carbon metabolism flux as protein acetylation stabilizes transcriptional and metabolic differences (25, 26). Ornithine and citrulline are involved in arginine metabolism that help to maintain cellular redox homeostasis (27), with cluster two strains showing increased levels of both ornithine and citrulline, reflecting an attempt to mitigate a rise in stress caused by faster growth rates (Fig. 2A and E). Elevated levels of methionine in cluster two strains also supported the maintenance of redox homeostasis (Fig. 2A) (28). In conclusion, DR strains of *M. tuberculosis* show broadly rewired metabolomes compared to the DS parent; however, most metabolite shifts were moderate and correlated with growth rather than being strong, pathway-specific shifts associated with individual resistance mutations (Fig. 2F).

### Diverse DR strains have increased sensitivity to propionate-induced toxicity

**Fig 3:**
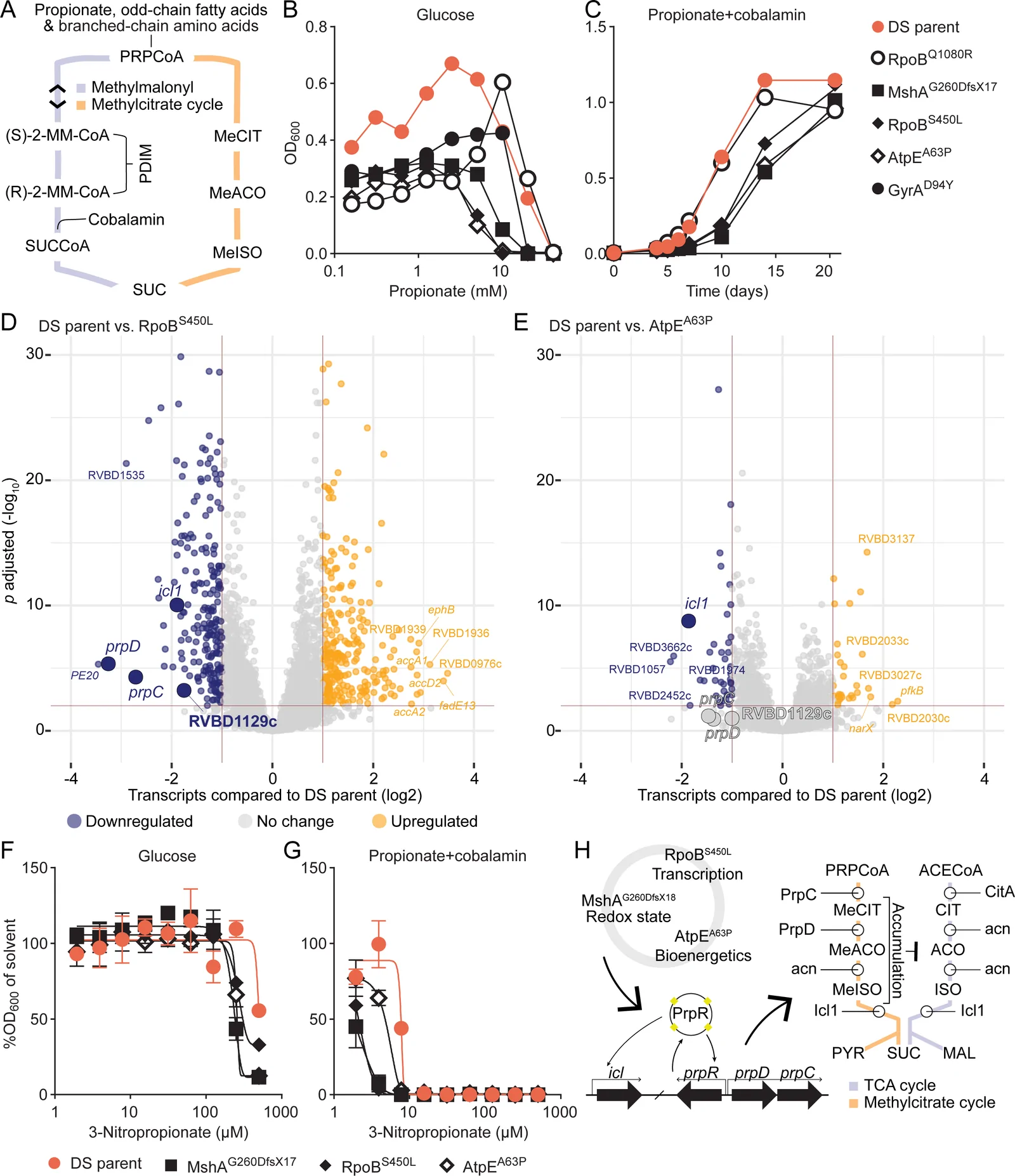
Propionate growth inhibition of DR strains is caused by dysregulation of propionate metabolism genes. (**A**) Propionate metabolism pathways, with methylmalonyl in blue, MCC in orange, and phthiocerol dimycocerosate (PDIM) representing methyl-branched lipids. (**B**) Minimum inhibitory concentration (MIC) assay of propionate in 7H12 + glucose. Circles show the average of two biological replicates, each conducted with two technical replicates. (**C**) Growth curves of DR strains on 7H12 propionate + cobalamin. Circles show the average of three biological replicates. Note: GyrA^D94Y^ was not included. (**D and E**) Volcano plots comparing the transcript levels of the DS parent to RpoB^S450L^ (**D**) and AtpE^A63P^ (**E**). Circles show the average log_2_ fold change in transcript abundance from five biological replicates. Red lines denote the thresholds: log_2_ fold change of >1 and <−1 and adjusted *P*-value of <0.01. Values not meeting these thresholds are colored gray, while significant values with a fold change of >1 are orange and <−1 are blue. Note: 10 genes with an adjusted *P*-value of <1 × 10^−30^ are not shown. (**F and G**) MIC assays of 3-nitropropionate in 7H12-glucose (**F**) and -propionate + cobalamin (**G**). Circles show the average of two biological replicates, each conducted with at least two technical replicates, and error bars show the range. (**H**) Model of propionate-induced toxicity, with the MCC in orange and the TCA cycle in blue. Black-outlined open circles represent proteins, and yellow diamonds represent an iron-sulfur cluster. Raw growth data for growth curves of DR strains on 7H12 propionate + cobalamin (**C**) are presented in Table S5. Gene expression data with associated significance values are presented in Table S6. Metabolites: PRPCoA, propionate-CoA; ACECoA, acetyl-CoA; Me, methyl group; CIT, citrate; ACO, aconitate; ISO, isocitrate; PYR, pyruvate; SUC, succinate; MAL, malate.

Propionate can be metabolized through three different pathways: the methyl-citrate cycle (MCC) that produces growth-inhibiting metabolites, the non-toxic cobalamin-dependent methylmalonyl pathway, and incorporation into methyl-branched cell wall lipids (Fig. 3A) (29, 30). The growth of DR strains RpoB^S450L^, AtpE^A63P^, MshA^G260DfsX18^, and GyrA^D94Y^ were inhibited on 0.1% (~10 mM) propionate in growth assays, while the typically poor-growing RpoB^R1080Q^ strain showed better growth on propionate than the DS parent (Fig. 1B). We hypothesized that these growth impairments were due to increased sensitivity to propionate-induced toxicity. We first confirmed this in dose response assays with RpoB^S450L^, AtpE^A63P^, MshA^G260DfsX18^, and GyrA^D94Y^, all showing at least a 2-fold increase in sensitivity to propionate (Fig. 3B). Propionate toxicity was induced through the MCC, as cobalamin supplementation, which enables propionate catabolism through the alternative methylmalonyl pathway, rescued the growth inhibition of 0.1% propionate in growth assays in these strains, although they still showed extended lag phases (Fig. 3C).

We hypothesized that increased propionate sensitivity was shared among genetically diverse strains because of overlapping dysregulation in propionate metabolism genes. To investigate this, we performed transcriptomic analyses of RpoB^S450L^ and AtpE^A63P^. Culturing was carried out in 7H9-OADC to parallel the conditions the strains were cultured in prior to being tested for growth in 7H12 media. Compared to the DS parent, RpoB^S450L^ exhibited 256 upregulated and 215 downregulated genes (Fig. 3D), while AtpE^A63P^ had just 29 upregulated and 39 downregulated genes (log_2_ fold change > 1 or < −1; adjusted *P*-value < 0.01) (Fig. 3E). Despite AtpE^A63P^ having a low number of dysregulated genes, it shared 10 upregulated genes and 24 downregulated genes with RpoB^S450L^. Of relevance to propionate metabolism, both strains had reduced expression levels of genes *prpR*, *prpDC*, and *icl1*, although only *icl1* was significantly downregulated in AtpE^A63P^ (Fig. 3D and E).

MCC-derived propionate toxicity occurs in part through the inhibition of isocitrate lyases (30). On this basis, we hypothesized that if reduced *icl1* expression increased sensitivity to propionate-derived toxicity, then propionate-sensitive strains should have increased sensitivity to Icl1 inhibitors, including 3-nitropropionate (31). Consistent with this, all propionate-sensitive DR strains were more sensitive to 3-nitropropionate compared to the DS parent when grown on either 7H12-glucose or 7H12-propionate + cobalamin (Fig. 3F and G). Consistent with the primary target of 3-nitropropionate, Icl1 being required for fatty acid catabolism, we observed increased efficacy of 3-nitropropionate when grown on propionate + cobalamin compared to glucose (~10×) (Fig. 3F and G), which is consistent with prior reports of 3-nitropropionate having enhanced growth inhibition when grown on pyruvate, lactate, or butyrate (32, 33). The low-level growth inhibition of 3-nitropropionate on glucose is the likely result of 3-nitropropionate inhibiting other enzymes, including succinate dehydrogenase and InhA, albeit with lower affinity (34, 35). In conclusion, increased propionate sensitivity in genetically diverse DR strains occurred via the MCC, with low transcription levels of *icl1* and other propionate metabolism genes likely causing MCC metabolites to accumulate and, in turn, impede the TCA cycle (Fig. 3H).

### Metabolic maladaptation of DR strains is the result of multi-gene dysregulation

**Fig 4:**
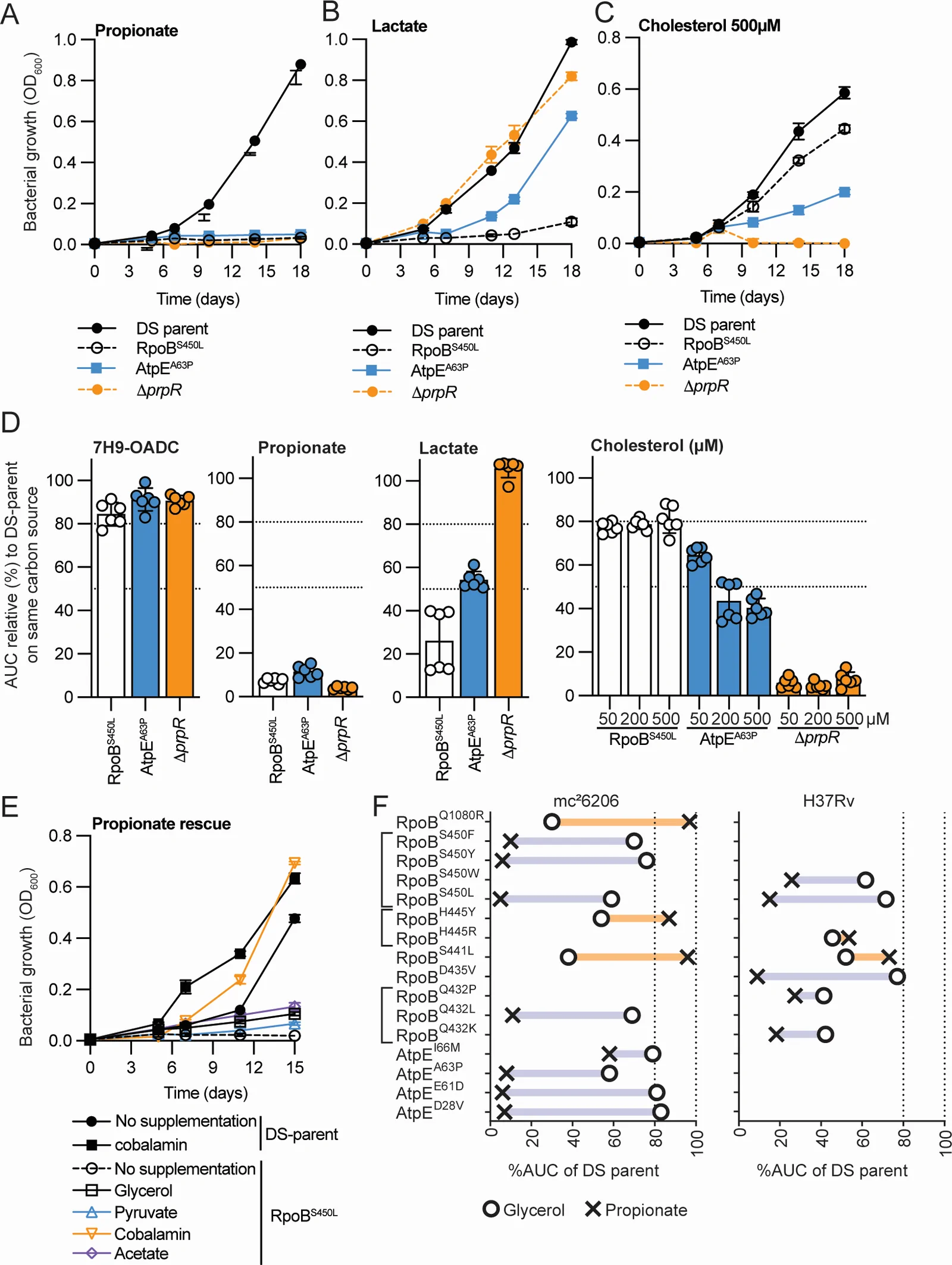
Propionate growth impairment extends to additional RpoB and AtpE mutants. (**A–C**) Growth curves of a DS parent, RpoB^S450L^, AtpE^A63P^, and Δ*prpR* on 7H12 supplemented with (**A**) propionate, (**B**) lactate, or (**C**) cholesterol (500 µM). Symbols show the average OD_600_ values from three biological replicates of representative experiments. (**D**) Percent AUC of RpoB^S450L^, AtpE^A63P^, and Δ*prpR* relative to the DS parent across different carbon sources. (**E**) Rescue of RpoB^S450L^ when grown in 7H12-propionate with additional carbon sources. In each case, mutants are compared to the DS parent grown on the same carbon source. Concentrations of supplemented carbon sources are comparable to those used in prior growth assays. (**F**) Percent AUC of mc^2^6206 RpoB and AtpE mutants and H37Rv RpoB mutants compared to DS parent on glycerol (open circles) and propionate (× symbols). Mutations present at the same RpoB amino acid are grouped. Blue lines indicate strains with lower growth on propionate, and orange indicates strains with higher growth. Symbols show the mean of biological triplicate assays. Raw growth data and AUC values with associated statistical analyses are presented in Table S5.

We hypothesized that if the metabolic maladaptation of DR strains was the result of broad transcriptional dysregulation, then DR strains should not phenocopy single deletion mutants of transcriptionally dysregulated genes, such as *prpR* and *icl1*. Consistent with this, RpoB^S450L^ and AtpE^A63P^ had a comparable growth defect to a Δ*prpR* mutant on propionate (Fig. 4A and D); however, the RpoB^S450L^ and AtpE^A63P^ growth impairment on lactate was not observed for Δ*prpR* (Fig. 4B and D). Cholesterol, a readily available lipid in a host setting, is metabolized into propionyl-CoA, with 2.6 mM of cholesterol being estimated to yield a concentration of propionyl-CoA equal to 0.5–1 mM. We hypothesized that the initial lack of a fitness cost of propionate-sensitive DR strains on cholesterol was due to the level of cholesterol used, i.e., 50 µM, generating insufficient propionyl-CoA to cause growth inhibition (~10 mM propionate used in growth assays) (36, 37). To test this, we re-evaluated fitness across increasing cholesterol concentrations. While the fitness cost for RpoB^S450L^ remained consistent across 50, 200, and 500 µM cholesterol (77%, 79%, and 80% relative to wild type, respectively, compared to 84% on fully supplemented 7H9), the fitness costs for AtpE^A63P^ increased in a dose-dependent manner (64%, 44%, and 40% relative to wild type) (Fig. 4C and D). In all cases, both strains grew better than Δ*prpR*, which failed to grow at any cholesterol concentration (Fig. 4C and D). These phenotypic differences demonstrate that the propionyl-CoA sensitivity of RpoB^S450L^ and AtpE^A63P^ is not strictly due to the dysregulation of *prpR*. Icl1, expression of which was dysregulated in both RpoB^S450L^ and AtpE^A63P^, functions in both the methylcitrate and TCA cycles. While *icl1* mutants have reciprocal growth impairments to RpoB^S450L^ and AtpE^A63P^ on propionate and lactate, the propionyl-CoA toxicity of RpoB^S450L^ can only be complemented by cobalamin, while the propionyl-CoA toxicity of *icl1* mutants can be rescued by cobalamin or acetate supplementation (Fig. 4E) (30, 38). In conclusion, the metabolic fitness costs of RpoB^S450L^ and AtpE^A63P^ are the result of a broad cellular dysregulation, rather than the dysregulation of an individual gene.

### Propionate sensitivity is not universally conserved among rifampicin- or bedaquiline-resistant mutants

Resistance to rifampicin and bedaquiline can be provided by various *rpoB* and *atpE* mutations. Since the RpoB^Q1080R^ mutant was not inhibited for growth on ~10 mM propionate like RpoB^S450L^, we hypothesized that propionate sensitivity would not be universal amongst different RpoB- or AtpE-resistant mutants. To investigate this, we constructed additional *rpoB* rifampicin-resistant genotypes (Q432L, S441L, H445Y, S450Y, and S450F) and bedaquiline-resistant *atpE* genotypes (D28V, E61D, and I66M). Growth assays revealed RpoB mutants S450L, S450Y, S450F, and Q432L had similar levels of growth on glycerol yet were unable to grow on propionate (Fig. 4F). Conversely, RpoB Q1080R, H445Y, and S441L had the lowest growth levels on glycerol yet had improved growth on propionate (Fig. 4F). Importantly, these observations translated into virulent H37Rv rifampicin-resistant mutants, with RpoB S450 and Q432 mutants showing impaired growth on propionate, while H445 and S441 mutants had better growth on propionate (Fig. 4B). Growth assays of the additional AtpE mutants demonstrated that D28V and E61D had significant growth defects on propionate, while I66M had a marginal growth impairment (Fig. 4B). In conclusion, propionate sensitivity extends to various RpoB and AtpE mutants in mc^2^6206 and virulent H37Rv but is not universally conserved.

### DR strains of *M. tuberculosis* have condition-specific collateral vulnerabilities

Previous work in *M*. tuberculosis has demonstrated that drug resistance causes altered sensitivity to functionally unrelated antibiotics (13). However, much of this work was carried out using 7H9-OADC or similar media. Given that we observed condition-specific growth defects, we speculated that DR strains would have condition-specific collateral vulnerabilities. Specifically, as RpoB and AtpE mutants shared increased sensitivity to propionate, we hypothesized that in the presence of propionate the RpoB mutants would be more susceptible to ATP synthase inhibition compared to the parent strain and, reciprocally, that the AtpE mutants would be more susceptible to RNA polymerase inhibition. To investigate this, we tested RpoB mutants for altered sensitivity to bedaquiline and TBAJ-587 and AtpE mutants to rifampicin and fidaxomicin when grown in 7H12-propionate + cobalamin versus -glucose.

**Fig 5:**
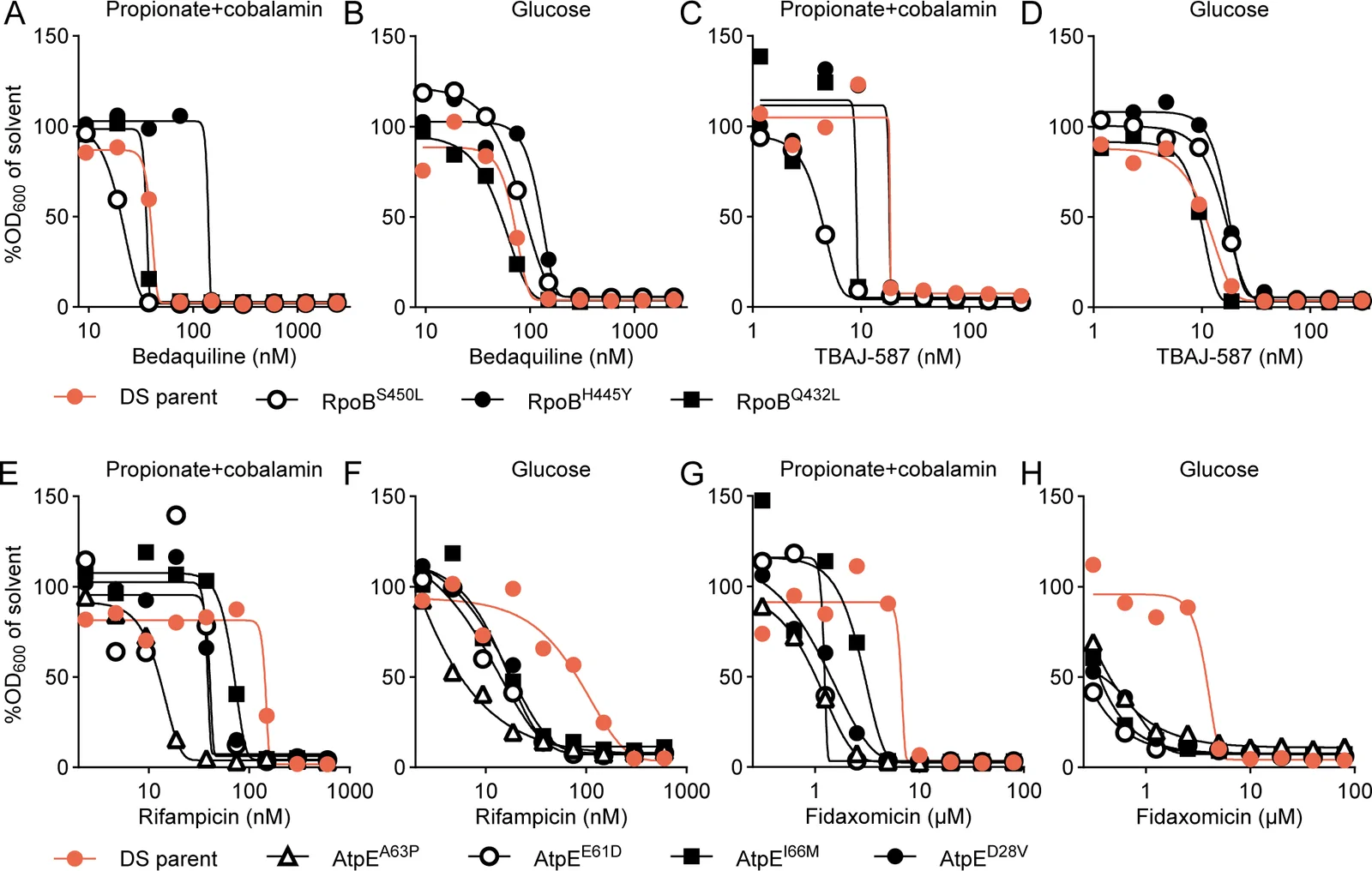
Altered antimicrobial susceptibility of mutant *rpoB* and *atpE* genotypes in the presence of propionate and cobalamin. (**A–H**) MIC assays of bedaquiline (**A and B**), TBAJ-587 (**C and D**), rifampicin (**E and F**), and fidaxomicin (**G and H**) in either 7H12-propionate + cobalamin or 7H12-glucose. Circles show the average of two biological replicates, each conducted with two technical replicates.

Consistent with propionate enhancing bedaquiline killing (39), bedaquiline had increased activity against the DS parent in 7H12-propionate + cobalamin compared to glucose (MIC of 45–97 nM) (Fig. 5A and B). This observation extended to RpoB^S450L^, which showed a greater shift in MIC against bedaquiline (31–148 nM) as well as a greater shift in MIC against TBAJ-587 (6–26 nM) compared to the DS parent (Fig. 5A through D). RpoB^H445Y^, which was able to grow on 10 mM propionate, showed no clear shift in MIC in any challenge (Fig. 5A through D), while RpoB^Q432L^, which was unable to grow on 10 mM propionate, exhibited an RpoB^S450L^-like MIC against TBAJ-587 on propionate + cobalamin (Fig. 5C).

The AtpE mutants were generally more susceptible to rifampicin and fidaxomicin compared to the DS parent in both media (Fig. 5E through H). However, only in 7H12-propionate + cobalamin were the MIC curves different among AtpE mutants (Fig. 5E through H), which corresponded to the level of growth inhibition on propionate (Fig. 4A). The greatest difference in MIC versus the parent being AtpE^A63P^ when challenged with rifampicin in 7H12-propionate + cobalamin (~8-fold, 159 versus 21 nM), compared to the AtpE^I66M^ mutant that had the lowest increase in sensitivity and had the lowest fitness cost on propionate (Fig. 5E through H). Overall, these data reveal that propionate metabolism, ATP production, and transcription are linked, with the simultaneous dysregulation of ATP production and RNA polymerase function being highly synergistic under host-relevant conditions. In conclusion, the altered metabolism of DR strains can lead to condition-dependent collateral vulnerabilities in *M. tuberculosis*.

## DISCUSSION

The host environment of *M. tuberculosis* is an ever-changing, hostile environment that *M. tuberculosis* must sense and adapt to in order to proliferate (40). Here, we demonstrate that antibiotic resistance alters fitness not only in a growth-optimized medium but also under different host-relevant conditions. In many cases, unrelated resistance mutations had overlapping phenotypes and metabolic dysregulation. These pleiotropic impacts on mycobacterial physiology limited the ability of DR strains to adapt to different environments and have profound implications for our understanding of how DR strains evolve within a clinical setting.

Propionate, branched-chain amino acids, and host-derived fatty acids (e.g., cholesterol) metabolism produce propionyl-CoA, the accumulation of which is toxic (41, 42). Propionyl-CoA metabolism through the MCC requires PrpDC as well as enzymes that are shared with the TCA cycle (Fig. 3H). Transcript and protein levels of *icl1* and *prpDC* are differentially upregulated in mycobacteria grown in propionate compared to glucose (33), with PrpR positively autoregulating its own transcription as well as the divergently transcribed *prpDC* genes and *icl1* (36, 43) (Fig. 3H). Here, we demonstrate that the sensitivity of RpoB^S450L^ and other DR strains to propionate toxicity is in part driven by reduced transcription of *icl1* and other propionate metabolism genes prior to condition switching. We conclude that these reduced transcription levels in rich media delay the metabolic shift to propionate catabolism from 7H9-OADC, causing MCC metabolites to accumulate and inhibit growth. The dysregulation of a single gene alone insufficiently explains the observed phenotypes of DR strains, demonstrating that fitness costs are the result of a broad cellular dysregulation.

The RpoB^S450L^ mutation affects RNA polymerase function by reducing transcription rates and having pro-pausing phenotypes (44–46). Conversely, alternative RpoB mutants exhibit increased transcription rates and have anti-pausing phenotypes (44–46). On this basis, the downregulation of *prpRDC* and *icl1* in RpoB^S450L^ in 7H9-OADC suggests that the expression of genes regulating propionate metabolism is either directly or indirectly affected by the impaired transcription of RNA polymerase containing RpoB^S450L^ (47). Supporting this, we observed contrasting propionate growth phenotypes among different RpoB mutants that are reported to have the opposite effect on transcriptional efficiency; for example, RpoB^H445Y^, which shows increased rates of transcription and anti-pausing, had improved growth on propionate (44, 48). Despite RpoB^S450L^ being the most fit mutant by frequency in clinic, accounting for >60% of all rifampicin-resistant clinical isolates, RpoB^S450L^ clinical isolates have high rates of compensatory mutations in *rpoC* to alleviate assumed fitness costs (49, 50). However, paradoxically, clinically less fit genotypes, including RpoB^H445Y^, have lower rates of compensatory mutations (49, 51). Based on the results presented here, we hypothesize that the reduced ability of RpoB^S450L^ to upregulate metabolic genes needed to respond to changes in nutrient availability would generate strong selective pressure to evolve compensatory mutations that restore transcriptional defects and metabolic gene expression (52). Conversely, selective pressures for compensatory mutations are likely to be absent, or weaker, in *rpoB* mutants that do not experience these metabolic defects (i.e., H445Y). The auxotrophies of mc^2^6206 are unlikely to confound the observed propionate phenotypes in DR mc^2^6206 strains because these phenotypes translated to equivalent DR H37Rv strains. In conclusion, metabolic defects in rifampicin-resistant mutants of *M. tuberculosis* are highly variable, which can limit their ability to adapt to changes in environmental conditions and possible evolutionary trajectories in clinical populations.

As part of the oral BPaL regimen, bedaquiline has excellent efficacy against DR strains in clinical settings. Under *in vitro* conditions, bedaquiline has increased efficacy against isoniazid-resistant mutants, suggesting that the efficacy of the BPaL regimen is in part the result of bedaquiline inadvertently targeting collateral vulnerabilities in strains with *katG*-mediated isoniazid resistance (13, 53, 54). Here, our results expand upon these observations by demonstrating that rifampicin-resistant RpoB mutants have condition-dependent collateral vulnerabilities to bedaquiline and TBAJ-587. Given that propionyl-CoA is derived from various host-relevant carbon sources and that rifampicin-resistant RpoB mutants are the predominant clinical resistance strains, we hypothesize that propionyl-CoA metabolism would generate collateral vulnerabilities in DR strains that could be exploited by bedaquiline. Further *in vivo* investigations of how different *rpoB* genotypes respond to treatment by bedaquiline or improved derivatives are required to investigate this and to determine if these condition-dependent collateral vulnerabilities are contributing to the positive clinical impact of bedaquiline. In conclusion, drug resistance can produce condition-specific collateral vulnerabilities, highlighting the importance of considering how physiological variation in *M. tuberculosis* affects the outcomes of new treatment regimens.

An unexpected observation from this study was the conservation of propionate sensitivity among different DR strains (Fig. 3). The transcriptional regulator of propionate metabolism, PrpR, binds a CoA ligand and contains a 4Fe-4S cluster that is thought to regulate activity in response to iron, as opposed to redox state (55). However, the increased sensitivity of MshA^G260DfsX18^ to propionate-induced toxicity suggests that intracellular redox state also plays a role in determining the activity of PrpR. The redox state of PrpR is likely dysregulated in MshA^G260DfsX18^, as mycothiol biosynthesis mutants exhibit higher numbers of oxidized protein cysteines (56). It is conceivable that PrpR also senses O_2_ and nitric oxide signals, analogous to the 4Fe-4S cluster of the transcriptional regulator WhiB3, which modulates propionate assimilation into fatty acids (57, 58). We found that expression of *prpRDC* and *icl1* was dysregulated in AtpE^A63P^, which is consistent with bedaquiline challenge altering Icl1 production (39) and inducing glucose flux through the reverse MCC, leading to a buildup of MCC metabolites (59). Moreover, the lack of Icl1 activity in the presence of propionate dysregulates the proton motive force and acidifies the cytoplasm in *M. tuberculosis* (42). The AtpE^A63P^ mutation, as well as propionate-sensitive E61D and D28V mutants, likely yield potentially defective ATP synthases that cause bioenergetic defects, requiring ATP synthesis through non-oxidative routes and increasing the membrane potential component of the proton motive force (52). Because of this, we hypothesize that propionate sensitivity in the AtpE mutants is the result of the toxicity of propionyl-CoA compounding the bioenergetic defects of defective ATP synthases. In conclusion, the regulation of propionate metabolism is likely modulated by complex interlinked bioenergetic and redox signaling, leading to diverse resistance mutants sharing propionate sensitivity phenotypes.

Propionate is catabolized through the MCC, methylmalonate cycle, and used for methyl-branched lipid production, while lactate and pyruvate drive the MCC in reverse (33, 42), with each of these carbon sources being relevant to in-host growth. Mutations in PrpR that delay growth on propionate-containing media provide antibiotic tolerance in clinical isolates (17), while cholesterol (which yields propionyl-CoA), pyruvate, and lactate are prominent in macrophages and granulomas (33, 60, 61). Despite the interconnected nature of these substrates, there were discrepancies in how DR strains grew on propionate, cholesterol, lactate, and pyruvate. Prior reports have demonstrated that pyruvate and lactate both induce the expression of *prpRDC* and *icl1*, although *icl1* is only required for growth on lactate, while *prpRDC* is dispensable under both conditions (33, 42). The lack of growth impairment on pyruvate for propionate- and lactate-sensitive DR strains could be a combination of resistance-induced transcriptional dysregulation and differences in genetic requirements under each condition. Alternatively, DR strains may be unable to upregulate lactate dehydrogenase to facilitate the conversion of lactate to pyruvate (40), or the fitness cost on lactate, but not pyruvate, may reflect a broader redox imbalance and increased sensitivity to reductive stress (62). This increased sensitivity to reductive stress is consistent with a *mshA* mycothiol-deficient mutant having identical growth impairments to both the RpoB and AtpE mutants (62). While RpoB^S450L^ mutants had only a minimal fitness cost at increasing concentrations of cholesterol, the AtpE^A63P^ mutant suffered from significant fitness costs at high cholesterol concentrations. The high levels of cholesterol in macrophages should yield correspondingly high levels of propionyl-CoA when catabolized by *M. tuberculosis* and allow these metabolic maladaptations to translate to infection-relevant conditions.

Within our study, there is the potential for confounding effects of background or secondary mutations. Many strains that contained a SigA^H425Q^ had increased growth on most media (e.g., RplC^C154R^, Gid^P75S^, MmpL3^F644C^, and Rv0678^G65GfsX10^), while *rrl*^a2269at^ generally showed notably low or delayed growth, suggesting that it was possible that SigA^H425Q^ contributed to these increased growth phenotypes (13). Conversely, prior work demonstrated that a SigA^H425Q^-containing strain caused no differences in growth (63), while the upregulation of *sigA* has been shown to improve growth of *M. tuberculosis* (64). Separately, in the absence of propionyl-CoA mutations in phthiocerol dimycocerosate, biosynthesis genes accumulate over successive passages, leading to altered growth and metabolic profiles (65). No trends in growth phenotypes on propionate were observed for strains with or without mutations in these genes (Table S7). Moreover, in this study, our semi-targeted MS identified methylcitrate, indicating the presence of propionyl-CoA, which was likely used for the production of phthiocerol dimycocerosates and selected against the loss-of-function mutations in their biosynthesis genes. Overall, we hypothesize that additional mutations did not confound the growth impairments observed in this study.

Here, we have demonstrated that the ability of *M. tuberculosis* to adapt to more host-like conditions is influenced by the acquisition of antibiotic resistance mutations. Genetic, metabolic, and environmental interactions in DR strains alter mycobacterial physiology and are pleiotropic, which have major implications for our understanding of how drug resistance evolves in a clinical context. Alterations in the physiology of DR strains can lead to condition-dependent collateral vulnerabilities, suggesting that antibiotics might be more effective against DR strains under certain host settings. Combined, these findings extend our understanding of the consequences of drug resistance mutations and how DR strains of *M. tuberculosis* evolve and adapt to a host environment.

## MATERIALS AND METHODS

### Bacterial strains and growth conditions

This study was conducted with *M. tuberculosis* strains H37Rv and mc^2^6206 (Δ*leuCD* and Δ*panCD*) and their derivatives. All *M. tuberculosis* strains used in this study are listed in Table S7. *M. tuberculosis* H37Rv cultures were maintained in 7H9 (BD, #271310) liquid medium supplemented with 0.05% tyloxapol (Sigma, #T8761) and OADC (0.005% oleic acid [Sigma, #O1008], 0.5% bovine serum albumin [pH Scientific, #PH100], 0.2% dextrose [Sigma, #G8270], and 0.085% catalase [Sigma, #02071]). *M. tuberculosis* mc^2^6206 cultures were maintained in 7H9-OADC liquid medium with the addition of 25 µg/mL pantothenic acid (Sigma, #21210) and 50 µg/mL leucine (Sigma, #L8000). Where indicated, fatty acid-free bovine serum albumin (Sigma, #A7906) was used. Growth on solid medium was facilitated with 7H11-OADC supplemented as above (excluding tyloxapol). Growth kinetics assays of *M. tuberculosis* mc^2^6206 strains on single primary carbon source media were carried out with 7H12 (17) (7H9 salts, 0.1% casamino acids [Gibco, #223050]) supplemented with 25 µg/mL pantothenic acid, 50 µg/mL leucine, 0.05% tyloxapol, and a carbon substrate (Table S8). All *M. tuberculosis* strains were grown at 37°C without shaking in T25 tissue culture flasks unless stated otherwise.

### Growth kinetics assays

*M. tuberculosis* mc^2^6206 cultures were pelleted by centrifugation at room temperature for 10 min at 3,220 × *g*, and the cells were washed in a volume equal to that originally added with 2× in 7H12 containing 0.05% tyloxapol but no carbon source. Washed cultures were diluted to an OD_600_ of 0.1 in 7H12 containing 0.05% tyloxapol, but no carbon source; then, 600 µL was added to 11.4 mL of the assay medium to achieve a final OD_600_ of 0.005. Culture OD_600_ absorbances were measured on days 4, 5, 6, 7, 10, 14, and 21 post-inoculum from 1 mL of culture dispensed into a cuvette. Measurements were ended prior to day 21 if the DS parent surpassed an OD_600_ of 1.5. The densities of undiluted cultures were measured until any culture surpassed an OD_600_ of 0.9, after which the OD_600_ absorbances were measured following a half dilution with the assay medium. The spectrophotometer was blanked using the assay medium.

AUC was used to calculate total growth across assay periods. AUC was calculated using the trapezoidal rule: for each consecutive pair of time points, the baseline (starting inoculum; OD_600_ = 0.005) was subtracted from each measurement, and the two baseline-corrected values were then averaged and multiplied by the time interval to give the area. Areas were summed across all intervals to give the AUC; day 0 was included in the calculation as OD_600_ = 0.005. Growth rate was calculated by dividing the change in OD_600_ between two time points by the change in days between those two time points, with the µMAX being the greatest rate across all time points after averaging replicates. Statistical analyses comparing the mean of the DS parent to each DR strain for each condition were calculated in GraphPad Prism (V11.0.0). Here, mixed-effects analyses were performed on all replicate data (matched across rows) with a normal distribution, and sphericity was assumed, and Dunnett’s multiple comparisons were tested. To guide follow-up studies, we established a pragmatic cutoff of within 20% of WT AUC as representing no fitness cost.

### Metabolomic analyses

Metabolite extractions of *M. tuberculosis* mc^2^6206 cultures and LC-MS analyses were carried out as previously stated (53). Briefly, cultures were grown to the mid-late exponential growth phase in 7H9-OADC before culture volumes equal to 5 mL at an OD_600_ of 1 were lysed by bead-beating in extraction buffer. Lysates were centrifuged and supernatants filtered through 0.2-µm filter columns. Six biological replicates were performed for each strain. Metabolites were separated and detected using a Vanquish Horizon UHPLC system coupled to an Orbitrap ID-X Tribrid mass spectrometer. Data were collected using Thermo Tracefinder (V 4.1), and their quality was assessed by the peak variation of pooled samples and internal standards. Samples were run alongside an in-house library of 550 polar metabolites, which allowed for a semi-targeted approach to peak assignment. Reported data were acquired from two separate experiments: one including the DS parent and seven DR strains, and the other including the DS parent and eight DR strains.

For semi-targeted analyses, metabolites were assigned to sample peaks in El-MAVEN by comparison to the reference metabolites. Peaks observed in extraction buffer-only samples at levels >20% of the DS parent were excluded. Identified metabolites were recorded as raw values from the AUC. Raw data were processed using the MetaboAnalystR package in R (V4.00) as follows. Each data set was processed individually in the statistical analysis (one-factor) pipeline. Here, metabolites present in fewer than 85% of samples were removed, remaining missing values were imputed using the knn_smp method, and values were median-normalized within the respective sample. Normalized metabolite values were then expressed as a proportion of the corresponding DS parent average. Both data sets, each now expressed as a proportion of the DS parent, were combined by shared metabolites. The Kruskal-Wallis test was used to identify which metabolites were most significantly different between strains.

For untargeted analyses, automated feature detection was carried out separately for both data sets using El-MAVEN (V0.12.1). Raw spectral data were first converted to mzML (32-bit) format and centroided with MSConvert (V3.0.23129). Sample peaks were aligned to PBQC sample 1 using the OBI-Warp method. Detection and group filtering were performed with the following settings: mass domain resolution, 5; lower limit intensity range, 10,000; minimum peak intensity, AreaTop, 1 peak >10,000; minimum quality, 50% peaks >0.6; and minimal signal/blank ratio, 75% peaks >5. Detected features with fewer than 70% of peaks classified as good peaks and peak max quality lower than 0.8 were removed. Remaining features were manually inspected for a normal distribution. Duplicate features and features that had more than one pooled sample control without a peak were removed. Each data set was processed individually in the MetaboAnalystR Statistical Analysis pipeline. Here, missing values were replaced using the ReplaceMin function, and then values were median-normalized and log transformed.

### Minimum inhibitory concentration assays

MIC assays were carried out with *M. tuberculosis* mc^2^6206 strains in 96-well flat-bottom plates with nine-point compound dilutions. Compounds were 2-fold serially diluted in the appropriate solvent and then added to 75 µL of medium in plate columns 2–11 (solvent only, low-to-high compound concentration, respectively) across plate rows B–G. One hundred and fifty microliters of medium was added to the remaining wells (sterility controls). Added compounds made up no more than 2% of the total well volume. Compound-containing wells were inoculated with 75 µL of OD_600_-adjusted culture (0.01) to achieve a starting culture density of OD_600_ 0.005. For MIC assays performed with 7H12, cultures were first washed as described in the growth kinetics assays. Culture OD_600_ densities were measured after 10 days for assays conducted in 7H9-OADC and 14 and 21 days for 7H12-glucose and 7H12-propionate. Measurements were carried out using a Varioskan Lux microplate reader (Thermo Fisher Scientific) after 96-well plates were shaken for 1–2 min at 800 rpm. Resulting OD_600_ values were normalized to the average absorbance of the sterility control wells by subtracting the average background absorbance. Where included, MIC curves were generated using non-linear regression (curve fit) with the Gompertz equation in GraphPad Prism. MICs were considered shifted between strains/conditions when the Gompertz model-calculated MIC was ≥2-fold different across multiple replicates.

### Generation of rifampicin mutants in *M. tuberculosis* H37Rv

*M. tuberculosis* H37Rv was inoculated to an OD_600_ of ~0.01 in five culture flasks by adding 100 µL of late log-phase culture to 10 mL of 7H9-OADC. Once reaching an OD_600_ of ~0.5 (10–14 days), the cultures were combined and centrifuged for 10 min at 3,220 × *g*. Cell pellets were resuspended in 1 mL of 7H9-OADC and then 10-fold serially diluted to 1 × 10^−7^. One hundred microliters of the undiluted culture and the first three dilutions were spread onto 7H11-OADC agar, in triplicate, containing 3×, 9×, or 27× MIC of rifampicin, and the last four dilutions were spread on agar containing DMSO (solvent). Colonies were counted after 28 days, and single colonies were inoculated into 7H9-OADC containing rifampicin at a concentration matching that on which they were isolated. The *rpoB* mutations of isolated colonies were identified as follows. gDNA was crudely prepared by heating 500 µL of culture at 95°C for 30 min, followed by centrifugation at 13,000 × *g* for 5 min and filtering 200 µL of lysate through a 0.22-µm filter. The rifampicin resistance-determining region of *rpoB* was PCR amplified using Phusion (New England Biolabs, #E0553L) DNA polymerase. The PCR mixtures (20 µL; 1× HF buffer, 0.2 µM forward [TGGTCCGCTTGCACGA] and reverse [ATGTAGTCCACCTCA] primers, 50 ng template DNA, 200 µM dNTP mix, 0.5 units of Phusion, and 3% DMSO) were thermocycled as follows: 1 min at 98°C, 10 s at 98°C, 15 s at 50 °C, 30 s at 72 °C, and 5 min at 72°C, with steps 2–4 cycled 36 times. PCR products were cleaned using the Zymo Clean & Concentrator (Zymo Research, #D4014) as directed by the manufacturer and sequenced by Sanger sequencing using the forward primer at Genetics Analysis Services, University of Otago. Mutations were determined by comparing the DNA sequences of the mutants with wild-type *rpoB* (Fig. S4).

### Construction of RpoB, AtpE*,* and *prpR* mutants with mycobacterial recombineering

mc^2^6206 mutants were constructed using mycobacterial recombineering as previously described (66). Briefly, *M. tuberculosis* mc^2^6206 was transformed with pKM461. mc^2^6206 with pKM461 was grown; anhydrotetracycline was then added to induce the Che9C annealase, and cells were made electrocompetent as described in the transformation of CRISPR interference plasmids section. For RpoB and AtpE mutants, 380 µL of electrocompetent cells were transformed with 2 µL of recombineering oligo (1 µg/µL) containing an *rpoB* or *atpE* mutation. The oligo sequences are presented in Table S9. Electroporated cells were recovered in 5 mL 7H9-OADC at 37°C for 48 h. Cells were harvested and plated onto supplemented 7H11-OADC agar containing rifampicin (0.5 µM) or bedaquiline (0.2 µM). After 3 weeks of incubation, colonies were isolated into 10 mL of 7H9-OADC containing 20 µg/mL kanamycin. Isolated cultures were screened for rifampicin or bedaquiline resistance using MIC assays. Genomic DNA was extracted from phenotypically positive isolates, and Sanger sequencing of PCR products was used to confirm the *rpoB* or *atpE* mutations. To construct the *prpR* deletion mutant, 380 µL of electrocompetent ATc-induced *M. tuberculosis* mc^2^6206 + pKM461 was transformed with 1 µL of *prpR* deletion oligo (1 µg/mL) with 200 ng of pKM496 (zeocin-resistant). The oligo sequences are presented in Table S9. Electroporated cells were recovered in 5 mL 7H9-OADC at 37°C for 48 h. Cells were harvested and plated onto supplemented 7H11-OADC agar containing zeocin (25 µg/mL). After 3 weeks of incubation, colonies were isolated into 10 mL of 7H9-OADC containing 25 µg/mL zeocin. Genomic DNA was extracted, and whole genome sequencing was used to confirm the deletion of *prpR*. To cure the strains of pKM461, the mutants were grown in 10 mL of 7H9-OADC to an OD of approximately 0.1. Cultures were diluted and plated onto 7H11-OADC agar containing 3% sucrose. After 2 weeks of incubation, colonies were isolated into 24-well plates containing 1 mL of 7H9-OADC. Once turbid, isolates were screened for both kanamycin and rifampicin sensitivity to confirm the loss of pKM461 and maintenance of rifampicin resistance. Genomic DNA was extracted from confirmed isolates, and mutations in *rpoB* were again confirmed by Sanger sequencing and whole genome sequencing.

### RNA sequencing

RNA-seq analysis was performed as previously described (53). Briefly, five replicates per strain were cultured for 3 days. Cells were then harvested by centrifugation and then frozen. Total RNA was extracted using Zymo-RNA Clean and Concentrator (Zymo, #R1019) and DNase treated with Turbo DNA-free kit (Invitrogen, #AM1907). DNA removal was confirmed by PCR. RNA quality and integrity were assessed using the Aligent 2100 Bioanalysis system following the manufacturer’s instructions. RNA samples were prepared using GenTegra RNA tubes (#GTR5025-S). Libraries were prepared with Illumina’s Stranded Total RNA Prep Ligation with Ribo-Zero Plus kit and sequenced on a NextSeq2000 (SeqCenter). Demultiplexing, quality control, and adapter trimming were performed with bcl-convert (V3.9.3). Clean reads were aligned with Bowtie2 (V2.4.5) and indexed with SAMtools (V1.16.1). Differential gene expression between the DS parent and each DR strain was calculated with DESeq2 (V1.40.1), with a DESeq2 FDR method (Benjamini-Hochberg [BH] procedure) applied. The reference genome used for RNA sequencing was *M. tuberculosis* strain mc^2^6206.

## Data Availability

In addition to raw data published with this paper, all data and experimental components are fully available and without restriction upon request. Whole genome sequencing data for the *prpR* mutant are avaliable at the NCBI-SRA with accession number PRJNA1529887, while RNA sequencing data generated in this study are avaliable at the NCBI-SRA with accession number PRJNA1530648.

## SUPPLEMENTAL MATERIAL

Supplemental Figures mbio.00244-26-s0001.docx Figures S1 to S4.

Table S1 mbio.00244-26-s0002.xlsx Raw data for Fig. 1.

Table S2 mbio.00244-26-s0003.xlsx Raw and normalized metabolite levels from semi-targeted MS.

Table S4 mbio.00244-26-s0004.xlsx Raw metabolite levels from untargeted MS.

Table S5 mbio.00244-26-s0005.xlsx Raw data for Fig. 4.

Table S6 mbio.00244-26-s0006.xlsx RNAseq outputs.

Supplemental Tables mbio.00244-26-s0007.docx Tables S3 and S7 to S9.

ASM does not own the copyrights to Supplemental Material that may be linked to, or accessed through, an article. The authors have granted ASM a non-exclusive, world-wide license to publish the Supplemental Material files. Please contact the corresponding author directly for reuse.
