# Supplementary material for: Altered physiology of drug-resistant Mycobacterium tuberculosis restricts adaptation to host-relevant conditions and produces condition-dependent collateral vulnerabilities
Source: mBio. 2026 Sep 24;17(10):e00244-26. doi: 10.1128/mbio.00244-26 (PMC13712800; doi:10.1128/mbio.00244-26)
Supplement: Supplementary file 1 [file mbio.00244-26-s0001.docx]

**Figure S1. Maximum growth rates of DR strains from growth kinetics assays.** Bars represent the percentage of the mean µMax in comparison to the DS parent strain (100%, red bars). Dashed lines show 80 and 120% of the DS parent µMAX. Raw growth data used to calculate µMAX is presented in Table S1.

**Figure S2. Carbon substrate-dependent differences in growth kinetics of select DR strains.** Growth curves showing the growth of select DR strains. Symbols show the mean OD_600_ values from representative experiments. Strain symbols are depicted beside the final panel. Raw growth data for all experiments are presented in Table S1.

**Figure S3. Clustering of untargeted MS data.** sPLS-DA plots generated using the MetaboAnalystR SPLSR.Anal function for experiments 1 (A) and 2 (B). Symbols show single replicates that are compared across each of the top 5 components. Raw metabolite levels of untargeted MS data in Table S4.

**Figure S4. Sanger sequencing results revealing rifampicin-resistance mutations in *M. tuberculosis* H37Rv.** The Template row shows the wildtype *rpoB* sequence and its translation (with indices present). The Sequencing results of different *rpoB* mutants that were aligned to the Template sequence are shown in subsequent rows. Nucleotide substitutions are highlighted in Salmon with associated amino acid changes also shown.
