# Supplementary material for: Altered physiology of drug-resistant Mycobacterium tuberculosis restricts adaptation to host-relevant conditions and produces condition-dependent collateral vulnerabilities
Source: mBio. 2026 Sep 24;17(10):e00244-26. doi: 10.1128/mbio.00244-26 (PMC13712800; doi:10.1128/mbio.00244-26)
Supplement: Supplementary file 2 [file mbio.00244-26-s0007.docx]

**Table S1. Growth assay raw, AUC, and µMAX values. See TableS1 file.**

**Table S2. Raw and normalized metabolite levels from semi-targeted MS. See TableS2 file.**

**Table S3. Culture OD_600_ values from metabolite extractions.**

| Experiment | Strain | Replicate (OD_600_) | | | | | |
| --- | --- | --- | --- | --- | --- | --- | --- |
|  |  | 1 | 2 | 3 | 4 | 5 | 6 |
| 1 | Ds parent | 0.85 | 0.9 | 0.9 | 0.75 | 0.85 | 0.95 |
| 1 | GyrA^D94Y^ | 0.8 | 0.85 | 0.8 | 0.8 | 0.85 | 0.7 |
| 1 | RplC^C154R^ | 1 | 1.05 | 1.1 | 1.15 | 1.05 | 1.15 |
| 1 | QcrB^T313A^ | 0.75 | 0.78 | 0.85 | 0.75 | 0.85 | 0.8 |
| 1 | Ddn^K103KfsX58^ | 0.7 | 0.83 | 0.8 | 0.75 | 0.8 | 0.8 |
| 1 | AtpE^A63P^ | 0.75 | 0.83 | 0.8 | 0.75 | 0.8 | 0.85 |
| 1 | Rv0678^G65GfsX10^ | 1 | 1.23 | 1.05 | 1.2 | 1 | 1.35 |
| 1 | MshA^G260DfsX18^ | 1.1 | 1.1 | 1.1 | 1.05 | 1 | 1.2 |
| 2 | Ds parent | 0.9 | 0.95 | 0.8 | 0.9 | 0.95 | 0.95 |
| 2 | RpoB^S450L^ | 0.85 | 0.8 | 0.75 | 0.85 | 0.8 | 0.9 |
| 2 | RpoB^Q1080R^ | 0.65 | 0.65 | 0.6 | 0.7 | 0.6 | 0.6 |
| 2 | *rrl*^2269insTT^ | 0.75 | 0.8 | 0.75 | 0.8 | 0.85 | 0.9 |
| 2 | *rrs*^C517T^ | 0.95 | 1 | 0.9 | 1.05 | 1.05 | 1.1 |
| 2 | Gid^P75S^ | 0.85 | 1.1 | 1.15 | 0.95 | 1.05 | 1.1 |
| 2 | FbiC^T707P^ | 0.75 | 0.75 | 1 | 0.8 | 0.75 | 0.75 |
| 2 | DprE1^Y314H^ | 0.7 | 0.7 | 0.75 | 0.7 | 0.7 | 0.7 |
| 2 | MmpL3^F644C^ | 1.15 | 1.15 | 1.2 | 1.1 | 1.05 | 1.25 |

**Table S4.** **Raw metabolite levels from untargeted MS. See TableS4 file.**

**Table S5. Growth assay data of additional RpoB and AtpE mutants. See TableS5 file.**

**Table S6. RNA-seq fold change analyses. See TableS6 file.**

**Table S7. Bacterial strains used in this study.**

| Strain | Parent strain | Resistance-associated mutation | Additional non-synonymous mutations | | Reference |
| --- | --- | --- | --- | --- | --- |
| mc^2^6206 | H37Rv | NA | | NA | (1) |
| RpoB^S450L^ | mc^2^6206 | *rpoB*^C1349T^ | | None | (2) |
| RpoB^Q1080R^ | mc^2^6206 | *rpoB*^A3239G^ | | None | (2) |
| GyrA^D94Y^ | mc^2^6206 | *gyrA*^G280T^ | | None | (2) |
| RplC^C154R^ | mc^2^6206 | *rplC*^T460C^ | | rv2566^C2591T^, *sigA*^C1275G^, *mas*^5862+C^, *ppe52*^G594A^, *rv0200*^553+G^ | (2) |
| *rrl*^2269insTT^ | mc^2^6206 | *rrl*^2269insTT^ | | *rv0885*^T41C^, *sigA*^C1275G^ | (2) |
| *rrs*^C517T^ | mc^2^6206 | *rrs*^C517T^ | | *mce1F*^C115T^, *dinG*^T1682G^, *ilvA*^G1006A^, *fadD*26^G128A^, *rv2566*^C2591T^, *sigA*^C1275G^ | (2) |
| Gid^P75S^ | mc^2^6206 | *gid*^G223A^ | | *fadD*26^G128A^, *rv2566*^C2591T^, *sigA*^C1275G^, lprk^442+T^ | (2) |
| FbiC^T707P^ | mc^2^6206 | *fbiC*^A2119C^ | | None | (2) |
| QcrB^T313A^ | mc^2^6206 | *qcrB*^A937G^ | | *echA9*^G827C^ | (2) |
| Ddn^K103KfsX58^ | mc^2^6206 | *ddn*^307delA^ | | None | (2) |
| AtpE^A63P^ | mc^2^6206 | *atpE*^G187C^ | | *rv1184*^T352A^, *pe_pgrs*13^A1172G^ | (2) |
| Rv0678^G65GfsX10^ | mc^2^6206 | *rv0678*^193delG^ | | *rv2566*^C2591T^, *sigA*^C1275G^, *ppe3*^C1126G^ | (2) |
| MshA^G260DfsX18^ | mc^2^6206 | *mshA*^778delG^ | | rv2566^C2591T^, *sigA*^C1275G^, *rv0200*^553+G^ | (2) |
| DprE1^Y314H^ | mc^2^6206 | *dprE1*^T940C^ | | *ppe3*^C1126G^, *idtB*^C756A^ | (2) |
| MmpL3^F644C^ | mc^2^6206 | *mmpL3*^A1931C^ | | *mkl*^1006-A^, rv2566^C2591T^, *sigA*^C1275G^,  *ppsC*^2858delC^ | (2) |
| RpoB^S450Y^ | mc^2^6206 | *rpoB*^S450Y^ | | *rv3239c*^G18R^, PE_PGRS9^E191G;T252A;N280D^, *ugpA*^T224A^, PE_PGRS6^A239G^, *ppsB*^I896N^, *yjcE*^V454A^, *esxQ*^A113P^, *rv0007*^181fs^, *rv2478c*^133fs^, *rv0958*^148+VG^, *idsA2*^R309G^ | This study |
| RpoB^S450F^ | mc^2^6206 | *rpoB*^S450F^ | | *esxQ*^A113P^, PE_PGRS9^E191G;T252A;N280D^, PE_PGRS6^A239G^, *ppsB*^I896N^, *rv3239c*^G18R^, *rv0007*^181fs^, *rv2478c*^133fs^, PE_PGRS58^166fs^, *rv2553c*^58+V^, *idsA2*^R309G^, *rv0958*^148+VG^ | This study |
| RpoB^H445Y^ | mc^2^6206 | *rpoB*^H445Y^ | | *esxQ*^A113P^, PE_PGRS9^E191G;N280D^, PE_PGRS6^A239G^, *ppsB*^I896N^, *rv3239c*^G18R^, *rv0007*^181fs^, *rv2478c*^133fs^, *rv2553c*^58+V^, *idsA2*^R309G^, *rv0958*^148+VG^ | This study |
| RpoB^S441L^ | mc^2^6206 | *rpoB*^S441L^ | | *rv3239c*^G18R^, *ppsB*^I896N^, *pks5*^F1436L^, *rv0007*^181fs^, *galU*^P51A^, PE_PGRS51^G540A^ | This study |
| RpoB^Q432L^ | mc^2^6206 | *rpoB*^Q432L^ | | *esxQ*^A113P^, *pks13*^D147Y^, PE_PGRS6^A239G^, PE_PGRS9 ^E191G;N280D^, *ppsB*^I896N^, *rv3239c*^G18R^, *rv2478c*^133fs^, *rv0007*^181fs^, *idsA2*^R309G^, *rv0958*^148fs^, *rv3645*^V174G^, *rv2553c*^58+V^, | This study |
| AtpE^D28V^ | mc^2^6206 | *atpE*^D28V^ | | *Rv0925c*^T2A^, *rv3239c*^G18R^, PE_PGRS6^A239G^, PE_PGRS9^N280D^, *ppsB*^I896N^, *rv0007*^181fs^, *rv2478c*^133fs^, *rv1575*^130fs^, *rv2553c*^58+V^, *rv0958*^148fs^ | This study |
| AtpE^E61D^ | mc^2^6206 | *atpE*^E61D^ | | *rv3239c*^G18R^, PE_PGRS6^A239G^, *rv0007*^181fs^, *rv2478c*^133fs^, *idsA2*^R309G^, *rv0958*^148+VG^, *rv2553c*^58+V^ | This study |
| AtpE^I66M^ | mc^2^6206 | *atpE*^I66M^ | | *esxQ*^A113P^, *rv0007*^181fs^, *rv2478c*^133fs^, *rv0958*^148+VG^, PE_PGRS6^A239G^, *ppsB*^I896N^, *rv3239c*^G18R^, *rv3645*^V174G^, *rv2553c*^58+V^ | This study |
| H37Rv | NA | NA | | NA |  |
| RpoB^S450L^ | H37Rv | *rpoB*^C1349T^ | | NA | This study |
| RpoB^S450W^ | H37Rv | *rpoB*^C1349G^ | | NA | This study |
| RpoB^H445R^ | H37Rv | *rpoB*^A1334G^ | | NA | This study |
| RpoB^S441L^ | H37Rv | *rpoB*^C1322T^ | | NA | This study |
| RpoB^D435V^ | H37Rv | *rpoB*^A1304T^ | | NA | This study |
| RpoB^Q432K^ | H37Rv | *rpoB*^C1294A^ | | NA | This study |
| RpoB^Q432P^ | H37Rv | *rpoB*^A1295C^ | | NA | This study |
| MshA^G260DfsX18^ + pCiargD | MshA^G260DfsX18^ | *mshA*^778delG^ | | rv2566^C2591T^, *sigA*^C1275G^, *rv0200*^553+G^ | This study |
| mc^2^6206 + pCiargD | mc^2^6206 | NA | | NA | This study |
| mc^2^6206 + pKM461 | mc^2^6206 | NA | | NA | This study |

Note: whole genome sequencing of strains developed in this study were compared to an in-house mc^2^6206 reference sequence in Geneious Prime. fs denotes frameshift, and + an insertion.

**Table S8. Carbon substrates used in this study.**

| Substrate | Solvent/diluent | Working concentration | Supplier | Catalogue |
| --- | --- | --- | --- | --- |
| D-Glucose | Water | 0.2% (w/v) | Sigma | G7021 |
| Glycerol | Water | 0.2% (v/v) | Fisher Scientific | AJA242 |
| Na Pyruvate | Water | 0.2% (w/v) | Sigma | P2256 |
| K Acetate | Water | 0.2% (w/v) | Fisher Scientific | BP364 |
| Na Succinate dibasic | Water | 0.2% (w/v) | Sigma | S2378 |
| Fumaric acid | Water | 0.2% (w/v) | Sigma | F19353 |
| Na L-Lactate | Water | 0.2% (w/v) | Sigma | L7022 |
| Na Propionate | Water | 0.1% (w/v) | Sigma | P1880 |
| Cholesterol | 1:1 tyloxapol:EtOH | 50 µM | Sigma | C75209 |
| Oleic acid | 1:1 tyloxapol:EtOH | 50 µM | Sigma | 031997-06 |

Note: NaOH was added to fumaric acid to aid in solubilisation, resulting in a pH of 6.8.

**Table S9. Oligo sequences used to construct chromosomal mutants.**

| Mutation | Oligo sequence (5′-3′) ^a^ |
| --- | --- |
| RpoB-S450Y | ACGCTCACGTGACAGACCGCCGGGCCCCAGAGCGTACAGTCGGCGCTTGTGGGTCAACCCCGACAGCGGG |
| RpoB-S450F | ACGCTCACGTGACAGACCGCCGGGCCCCAGCGCAAACAGTCGGCGCTTGTGGGTCAACCCCGACAGCGGG |
| RpoB-H445Y | ACCGCCGGGCCCCAGCGCCGACAGTCGGCGCTTGTAGGTCAACCCCGACAGCGGGTTGTTCTGGTCCATG |
| RpoB-Q432L | CAACCCCGACAGCGGGTTGTTCTGGTCCATGAATAGGCTCAGCTGGCTGGTGCCGAAGAACTCCTTGATC |
| RpoB-S441L | CAGCGCCGACAGTCGGCGCTTGTGGGTCAACCCCAACAGCGGGTTGTTCTGGTCCATGAATTGGCTCAGC |
| AtpE-D28V | GACACCGGAGATAAGCGCGTTACCGGCGACACCGACACCGATACCGGCGCCGATGGCGCCACCGGCCAT |
| AtpE-E61D | CGCCATAAACGCCAGGTTGATGAAGTATGCCGCATCAACCAAACCGACGGTGATGAAGAACGGTGTGAAC |
| AtpE-I66M | AGCGAAGACGAACAGCGCCATAAACGCCAGGTTCATGAAGTATGCCGCCTCAACCAAACCGACGGTGATG |
| *prpR* deletion mutant | TTTGTGTCCTGATCCGGCACATTTTGCAAATTTTGCGAACATGACGCGGAGTAATGTCTTACCGGTGGCTGGTTTGTCTGGTCAACCACCGCGGTCTCAGTGGTGTACGGTACAAACCCCTTATTCGTCGACCGAGCAATCGGTTTGACCGCCCGACGCCACAGCAGACAACGAAACCCCTTATATTA |

**References**

1.	Jain P, Hsu T, Arai M, Biermann K, Thaler DS, Nguyen A, et al. Specialized transduction designed for precise high-throughput unmarked deletions in *Mycobacterium tuberculosis*. mBio. 2014;5(3):e01245-14.

2.	Waller NJE, Cheung CY, Cook GM, McNeil MB. The evolution of antibiotic resistance is associated with collateral drug phenotypes in *Mycobacterium tuberculosis*. Nat Commun. 2023;14(1):1527.
